# Direct Oral Anticoagulants versus Vitamin K Antagonists in Individuals Aged 80 Years and Older: An Overview in 2021

**DOI:** 10.3390/ijerph20021448

**Published:** 2023-01-13

**Authors:** Chana Azzoug, Gilles Nuémi, Didier Menu, Emmanuel De Maistre, Mathieu Boulin, Alain Putot, Patrick Manckoundia

**Affiliations:** 1 “Pôle Personnes Âgées”, Hospital of Champmaillot, University Hospital, 21079 Dijon, France; 2 Medical Information Department, University Hospital, 21079 Dijon, France; 3 “Mutualité Sociale Agricole” of Burgundy Franche Comté, 21000 Dijon, France; 4 Haemostasis Unit, University Hospital, 21079 Dijon, France; 5 Pharmacy Department, University Hospital, 21079 Dijon, France; 6 Department of Internal Medicine and Infectious Diseases, Pays du Mont Blanc Hospital, 74700 Sallanches, France; 7 Physiopathologie et Épidémiologie Cérébro-Cardiovasculaires (PEC2), EA 7460, University of Burgundy, 21000 Dijon, France; 8 INSERM U-1093, Cognition, Action and Sensorimotor Plasticity, University of Burgundy, 21000 Dijon, France

**Keywords:** aged 80 and over, anticoagulant, direct oral anticoagulants, vitamin K antagonists

## Abstract

Two main types of oral anticoagulants are available in France: vitamin K antagonists (VKA) and, more recently, direct oral anticoagulants (DOAC). The benefit–risk profile appears to be favorable for DOAC, which is as effective as VKA but safer (fewer cases of severe and cerebral bleeding). In a study in 2017, we observed that older adults did not seem to receive the same modalities of oral anticoagulants as younger individuals for various reasons. To assess anticoagulation prescribing practices over time, we repeated this cross-sectional study by comparing very old individuals taking DOAC to those taking VKA. Ambulatory individuals aged 80 years and older were included. They were affiliated with the Mutualité Sociale Agricole of Burgundy and were refunded for a medical prescription of oral anticoagulation in March 2021. The demographic characteristics, registered chronic diseases (RCD), number and types of prescribed drugs, and mortality of the DOAC group and the VKA group were compared. A total of 4275 subjects were included in the study: 67.44% (2883) received DOAC and 32.56% (1392) received VKA. The two groups were similar in age. In the DOAC group, there were more women (54.98% vs. 46.98%) (*p* < 0.001), fewer RCD (91.47% vs. 93.68%) (*p* = 0.014), and lower rates of venous thromboembolism (2.53% vs. 6.75%) (*p* < 0.001), severe heart failure (56.50% vs. 68.03%) (*p* < 0.001), and severe kidney diseases (1.38% vs. 3.59%) (*p* < 0.001), but there were more subjects with Alzheimer’s disease (7.49% vs. 4.31%) (*p* = 0.001). Individuals in the DOAC group had fewer prescriptions of furosemide (48.53% vs. 55.75%) (*p* < 0.001) and fibrates (2.32% vs. 3.88%) (*p* = 0.044). They also had more prescriptions of proton pump inhibitors (43.95% vs. 39.44%) (*p* = 0.006) and antirheumatics (1.60% vs. 0.65%) (*p* = 0.009) than those in the VKA group. There was no difference in mortality. This study revealed that prescribing practices for DOAC have changed over time.

## 1. Introduction

In France, people aged 75 years and older account for a continually increasing share of the population, reaching 9.2% in 2018 [1]. This growing rate of older individuals, due in part to the increase in life expectancy, can also be seen worldwide [2]. In these older individuals, cardiovascular and cerebrovascular events result in a higher rate of long-term disability and dependence [3,4]. Age is a significant risk factor for stroke and thromboembolism as well as for bleeding, particularly in frail older adults.

These complications are a major public health issue, and it is, thus, essential to use treatments that are both effective and safe. Anticoagulation is used preventively and curatively for several cardiovascular diseases. There are two main types of oral anticoagulation treatments: vitamin K antagonists (VKA) and direct oral anticoagulants (DOAC) [5]. The anticoagulant effect of VKA is achieved by the inhibition of the vitamin K epoxide reductase complex subunit 1 in the liver [6]. VKA have been used in patients with atrial fibrillation (AF) and venous thromboembolism (VTE) since the 1940s [7,8]. In France, the VKA that are widely used include fluindione, warfarin and, more rarely, acenocoumarol [9]. In 2008, four DOAC came on to the European market following their approval for the treatment of non-valvular AF (NVAF) and VTE. DOAC directly inhibit specific coagulation factors. Dabigatran inhibits thrombin (factor IIa), whereas apixaban, rivaroxaban, and edoxaban (not available in France) inhibit activated factor X (Xa) [10].

DOAC and VKA have similar indications, including stroke prevention in AF [11], curative treatment of VTE, and prevention of VTE recurrence [12]. Yet, there are also some notable differences between the two treatments. DOAC are not recommended in valvular AF, while they are specifically indicated in the prevention of VTE after surgery for total hip or knee replacement [12]. VKA are recommended in the prevention of thromboembolic complications in mitral valvular AF and prosthetic heart valve or thrombosis treatment in antiphospholipid syndrome [13]. Since 2016, and in the absence of contraindications, the European Society of Cardiology (ESC) and the European Heart Rhythm Association have recommended the use of DOAC rather than VKA when there is a need for oral anticoagulation in patients with diagnosed NVAF [14]. These two treatments are equally effective [15], but the risk of major bleeding is reduced with DOAC [16]. Similarly, DOAC have shown comparable efficacy and a significantly lower bleeding risk than warfarin in patients with acute symptomatic VTE [17]. Other studies have even found that DOAC have greater efficacy for reducing strokes, systemic embolic events, and deaths in older individuals with fewer intracranial hemorrhages [18]. During the early years of DOAC, they were not widely used in the older population, as shown by Manckoundia et al. [19], who compared the prescription rates of DOAC and VKA in ambulatory older adults. Their work suggested that physicians, who prescribed DOAC less frequently than VKA, were reticent to prescribe new drugs to older patients. The authors also showed that older patients were treated differently, which could be explained by a lack of hindsight, leading prescribers to err on the side of caution, and by the absence of an antidote.

The observed attitude towards the prescription of DOAC in our 2017 study prompted us to conduct a follow-up study four years later using the same design. Our aim was to ascertain whether physicians’ practices have changed with an increasing prescription of DOAC, especially since the latest guidelines of the ESC have confirmed [20] that DOAC should be the first-line treatment for NVAF. We repeated this study to compare the prescription rates of DOAC and VKA in ambulatory older adults and to assess the characteristics of included individuals.

## 2. Methods

### 2.1. Design of the Study

The present cross-sectional study used data collected over one month, between 1 March and 31 March 2021, from the Mutualité Sociale Agricole de Bourgogne (MSAB) database, a French regional agricultural health insurance agency. This work was performed in accordance with the Declaration of Helsinki and the French national standards.

### 2.2. Subjects

The study sample consisted of all MSAB-affiliated individuals aged ≥80 years living in Burgundy who were refunded for a prescribed oral anticoagulant during the study period. These were the sole inclusion criteria. There were no exclusion criteria.

Two groups were constituted, one composed of individuals with a DOAC prescription (DOAC group) and the other made up of individuals with a VKA prescription (VKA group).

Our institutional Ethics Committee was consulted and approved this observational study, which had no impact on patient management.

### 2.3. Collected Data

For each individual, age, gender, prescription duration of the oral anticoagulant, and the medical specialty of the prescribing physician were collected. In addition, we recorded registered chronic diseases (RCD) according to the International Classification of Diseases, 10th Revision [21]. The French public health system offers total coverage for treatment of diseases that are qualified as severe and chronic, which include RCD. We were able to collect RCD because they are declared to health insurance companies, generally by the patients’ general practitioner (GP) or medical specialist. In order to see the list of RCD, please refer to the method section of the article by Manckoundia et al. [19]. We also recorded the number of drugs per prescription and types of treatment prescribed at the same time as the anticoagulant. Finally, it was recorded if the individual had subsequently died.

For a given subject, DOAC or VKA prescription was considered novel if (1) it was made within three months preceding the date of inclusion, and (2) no DOAC or VKA prescription was found more than three months before the inclusion period. The three-month period was chosen because, in France, a prescription for a drug from list 1 (which includes DOAC and VKA) remains valid for a maximum of three months. List 1 includes drugs that can be toxic under normal conditions of use. These drugs are only delivered upon the presentation of a medical prescription and for the duration specified on the prescription.

Finally, we determined from the declared RCD whether the indication for the oral anticoagulation was AF or VTE. We limited the indications recorded to these two events as they are the only common indications for both DOAC and VKA.

### 2.4. Statistical Analysis

Means and standard deviations were used to describe quantitative variables, whereas numbers and percentages were used to describe qualitative variables. The two groups (DOAC and VKA) were compared in terms of mean age, age range, sex, existence of one or more RCD, mean number of RCD, mean number of drugs per prescription, anticoagulant prescription duration (novel prescription or refill), specialty of the prescriber, frequencies of AF and VTE, rate of selected RCD, and cardiovascular drugs. Using a bivariate analysis, the data were compared using the analysis of variance for quantitative variables and the χ^2^ test or the Fisher’s exact test for qualitative variables. A *p* value of <0.05 was considered significant. To analyze the association between the type of oral anticoagulant prescribed and each variable, a bivariate analysis using logistic regression was carried out, with the calculation of odds ratios (OR) and 95% confidence intervals (95% CI). A multivariate analysis using stepwise logistic regression was then performed. The multivariate analysis included variables for which at least one of the sizes of the two groups was greater than 10 and which, in addition, responded to multicollinearity.

The R Core Team (2019) software (R Foundation for Statistical Computing, Vienna, Austria, version 3.6.1) was used to conduct all statistical analyses [22].

## 3. Results

Altogether, 4275 subjects, 52.37% of whom were women and with a mean age of 87.7 years (range 80 to 104), had a prescription for anticoagulants. The DOAC group included 2883 individuals (67.44%) and the VKA group included 1392 individuals (32.56%). In the total sample size, 1854 (43.39%) individuals had AF and 167 (3.92%) had VTE.

Table 1 summarizes the baseline patient characteristics for the two treatment groups. There was no significant difference in age: patients on VKA were aged 87.86 ± 4.64 and those on DOAC were 87.59 ± 4.52 years (*p* = 0.075). There were significantly more women in the DOAC group than in the VKA group, 54.98% vs. 46.98% (*p* < 0.001). The mean number of RCD was significantly higher in the VKA group than in the DOAC group, 2.05 ± 1.21 and 1.78 ± 1.12 (*p* < 0.001). There were significantly more individuals with ≥1 RCD in the VKA group than in the DOAC group, 93.68% vs. 91.47% (*p* = 0.014). There was a significant difference in the mean number of drugs per prescription, 7.40 ± 2.80 for the DOAC group and 7.69 ± 2.98 for the VKA group (*p* = 0.002). Oral anticoagulation was mostly newly initiated, in 66.11% of cases in the DOAC group vs. 19.61% in the VKA group, whereas there were significantly more refill prescriptions in the VKA group than in the DOAC group, 80.39% vs. 33.89% (*p* < 0.001). GPs were the main prescribers overall, but there were significantly fewer GP prescribers in the DOAC group than in the VKA group, 86.78% vs. 92.03% (*p* < 0.001). There were significantly fewer individuals with AF in the DOAC group than in the VKA group, 41.55% vs. 47.2% (*p* = 0.001). The rate of VTE was also significantly lower in the DOAC group than in the VKA group, 2.53% vs. 6.75% (*p* < 0.001). There was no significant difference in mortality, the rate of which was 8.60% in the DOAC group vs. 9.41% in the VKA group (*p* = 0.415). There were significantly more patients who changed their anticoagulation treatment from VKA to DOAC than vice versa, 8.60% vs. 1.14% (*p* < 0.001).

In the DOAC group, apixaban was most commonly prescribed (64.34%, N = 1855), followed by rivaroxaban (24.77%, N = 714), and dabigatran (10.89%, N = 314). For the VKA group, fluindione was most commonly prescribed (60.92%, N = 848), followed by warfarin (35.56%, N = 495) and then acenocoumarol (3.52%, N = 49).

Table 2 compares the RCD in both groups using bivariate logistic regression. Alzheimer’s disease was more common in the DOAC group than in the VKA group (*p* < 0.001). Compared to the DOAC group, significantly more individuals in the VKA group had severe heart failure or heart rhythm disorders (*p* < 0.001), severe hypertension (*p* < 0.001), severe chronic nephropathy (*p* < 0.001), severe chronic respiratory failure (*p* = 0.021), and polypathology (illness not on the list) (*p* < 0.001). There was no significant difference between the two groups for active chronic liver diseases and cirrhosis, or diabetes.

Table 2 also compares the prescriptions of medications in the DOAC group and the VKA group using bivariate logistic regression. Furosemide (*p* < 0.001), digoxin (*p* = 0.005), fibrates (*p* = 0.006), statins (*p* = 0.015), nitrate derivatives (*p* = 0.019), and calcium channel blockers (*p* = 0.043) were significantly more frequently prescribed in the VKA group, while other antiarrhythmic drugs (*p* = 0.035), proton pump inhibitors (PPI) (*p* = 0.006), and antirheumatics (*p* = 0.009) were significantly more frequently prescribed in the DOAC group. Thirty-two subjects had a prescription for both a VKA and a DOAC.

The multivariate analysis showed that AF (*p* = 0.011), Alzheimer’s disease (*p* = 0.001), switching anticoagulation (*p* = 0.003), PPI (*p* = 0.015), and antirheumatics (*p* = 0.016) were significant determining factors for the prescription of DOAC vs. VKA, while male sex (*p* = 0.003), refill prescriptions (*p* = 0.000), GP prescribers (*p* = 0.144), VTE (*p* < 0.001), severe heart failure or heart rhythm disorders (*p* = 0.001), RCD (*p* = 0.036), severe kidney failure (*p* < 0.001), and current treatment with furosemide (*p* = 0.001), fibrates (*p* = 0.004) and heparins (*p* = 0.016) were significant factors for VKA prescription (see Table 3).

## 4. Discussion

Four years after Manckoundia et al. published their first study on the subject [19], we conducted a study with the same design to analyze the prescribing practices for oral anticoagulation in older adults. Similar to the first study [19], real-life data were obtained from a health insurance agency database. In addition, we collected mortality data.

**Figure 1:**
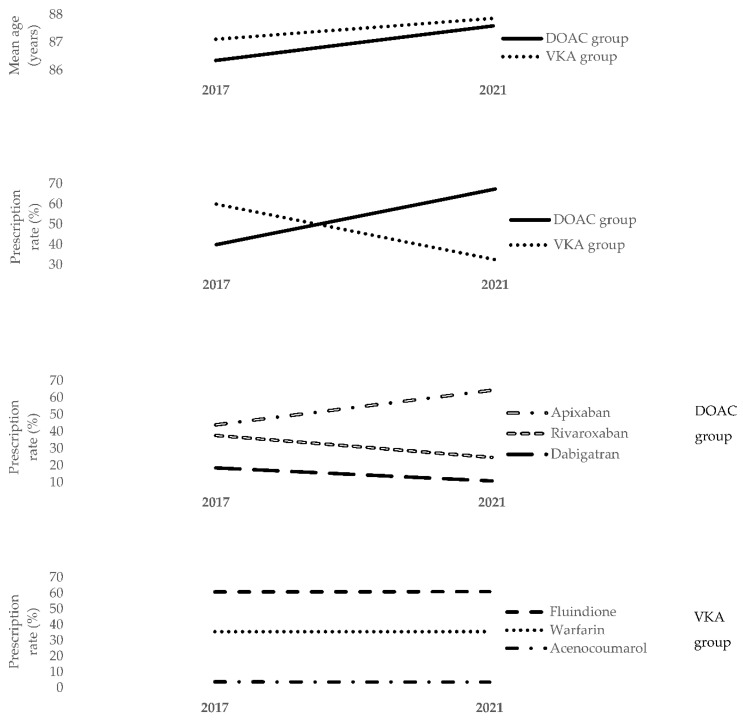
Comparison of the 2017 and 2021 studies with regard to the mean ages of the DOAC group vs. the VKA group; the rates (%) of DOAC vs. VKA; the rates (%) of apixaban, rivaroxaban, and dabigatran; and the rates (%) of fluindione, warfarin, and acenocoumarol.

The study sample consisted of 4275 individuals, with a mean age of 87.7 years, and including a majority of women (52.37%). The mean age, which was very advanced, was the same in the two groups, (87.58 in the DOAC group vs. 87.85 years in the VKA group), as were the proportions in the different age categories. The mean ages of the overall sample in the present study were almost identical (with a difference of a few months to a year) to those reported in the 2017 study [19] (See Figure 1).

In the present study, DOAC were prescribed twice as often as VKA. In 2017, Manckoundia et al. found that, despite the guidelines recommending that DOAC should be prescribed for the management of AF, the prescription rate of VKA remained considerably higher (one and a half times more than DOAC) (See Figure 1), probably because GPs were more reluctant to prescribe new treatments to older adults [19]. The trend observed herein has also been reported in other parts of the world; for example, the All Nippon AF In the Elderly (ANAFIE) registry conducted in Japan showed that 92.4% of patients were receiving DOAC from October 2016 to January 2020 [23]. To explain the prescription crossover between DOAC and VKA, it would have been interesting to have the age of the physicians, to know if the patients changed their GP, or to have other key information. While some of the participants analyzed here were also in the previous database, the current sample is not the same as in the previous study, as evidenced by a stable average age in the two study populations. The patient–physician pairs are, therefore, not the same, and it seems difficult to assess the impact of age or other characteristics of a given physician on the course of oral anticoagulant prescription for a given patient during the last four years.

In the present study, we also observed that VKA treatment was switched to DOAC in a number of individuals (8.68%). We can hypothesize that this suggests that practices are changing as confidence in DOAC increases. However, a prescription switch may also be due to the occurrence of an adverse event or changes in the patient’s health, among other reasons.

The shift in prescribing practices is likely a result of the current guidelines published by the ESC [20], which confirm that DOAC should be used as a first-line treatment in the absence of contraindications, and the publication of articles confirming that DOAC have a better benefit–risk ratio than VKA in older patients [24]. It seems that four years were long enough for prescribers, mostly GPs, to gain experience with these treatments, and to observe firsthand their efficacy and their relatively low risk. Physicians were most likely able to gain more insight and feel more comfortable as they noticed less bleeding, especially serious bleeding, in their own practice. The cost of the drug was not a factor in this study because, in France, treatments are reimbursed in full when they are linked to a RCD [25].

Furthermore, it seems that the patients who had their prescription switched to DOAC were satisfied, while still being concerned about side effects [26]. Despite the reversal of the trend from the findings of the study published in 2017, with DOAC now being the most commonly prescribed drugs, the rate of VKA prescription still appeared to be high, accounting for 32.56% of all prescriptions. This could be explained by possible contraindications to DOAC, the caution required in cases of kidney impairment, and the reluctance of patients to take or professionals to prescribe DOAC without implementing monitoring. Because, contrary to what we might think, the absence of biological tests does not always seem to be a factor promoting adherence to treatment [27]. It would have been interesting and instructive to know exactly why VKA was maintained in some cases, even though the current guidelines state that DOAC is preferred in the absence of contraindications [18,20]. Unfortunately, we did not have access to exhaustive medical files. We were, thus, unable to obtain diagnoses that were not RCD or the results of biological examinations since these data are not contained in the health insurance database that was used.

In the VKA group of our study, there were more refill prescriptions (80.39%) than novel prescriptions. This could be explained by the fact that GPs were by far the most frequent prescribers. In the DOAC group, there were more novel prescription (66.11%), confirming that the current trends have reached GPs, resulting in VKA being initiated less often.

Unexpectedly, 32 individuals had a prescription for both a VKA and a DOAC, which is a prescription error. These cases might be an oversight by the prescribing physician who, in wanting to switch one type of oral anticoagulant for another, prescribed both.

As in the 2017 study [19], apixaban was the most prescribed DOAC drug, followed by rivaroxaban and dabigatran. It is worth noting that the apixaban prescription rate increased by 20% between 2017 and 2021, while that of the other drugs decreased. The VKA drugs were prescribed similarly to the 2017 study [19]: fluindione was the most prescribed, followed by warfarin and acenocoumarol. Unlike DOAC, the prescription rates for these three VKA remained strictly the same between 2017 and 2021 (See Figure 1).

Concerning the indications for anticoaluation, AF was identified in 41.55% of the DOAC group and 47.20% of the VKA group, while VTE was found in 2.53% of the DOAC group and 6.75% of the VKA group. About half of the indications for oral anticoagulation are not known in the study, which is due to the fact that indications which are not RCD-related (non-serious VTE) or not declared as RCD by the patient’s physician are not available in our data. We were surprised to not find mechanical heart valves, for which there is an indication for long-term anticoagulation by VKA, and, to a lesser extent, bioprosthetic heart valves, for which the coverage for anticoagulant therapy is limited to three months [28].

In both groups, most patients had more than one RCD. However, compared to the VKA group, a higher proportion of patients in the DOAC group had no RCD (8.53% vs. 6.32%). The mean number of drugs was seven in both groups in 2021, confirming the trend for polypharmacy in the older population [29], while the mean number of drugs was five in 2017. In addition to the lower rate of RCD, the DOAC group had a significantly lower rate of severe comorbidities, such as severe heart failure or heart rhythm disorders (56.50% vs. 68.03%) and severe kidney diseases (1.38% vs. 3.59%), as confirmed in the multivariate analysis. Contrary to the 2017 study, severe hypertension and severe chronic respiratory failure were not the determining factors for the prescription of one of these drugs in the present study. Meanwhile, the patients in the DOAC group had higher rates of Alzheimer’s disease, probably because DOAC are easier to take since they do not require biological monitoring. As expected, severe chronic nephropathy was a factor associated with VKA prescription, as the French National Authority for Health (Haute Autorité de Santé) recommends the use of VKA rather than DOAC in patients with severely reduced renal function (i.e., creatinine clearance < 15 mL/min) [9]. It is known that kidney excretion of oral anticoagulants varies according to the DOAC used. The rate of kidney excretion is 27%, 33%, and 80% for apixaban, rivaroxaban, and dabigatran, respectively [30]. A dose reduction is needed for DOAC in certain situations: apixaban should be prescribed at five milligrams per day if two of the following characteristics are present: age ≥ 80 years, weight ≤ 60 kg, and creatinine ≥ 1.5 milligrams/deciliters. Rivaroxaban is prescribed at 15 milligrams per day to lessen the risk of major bleeding. Dabigatran is strictly contraindicated when creatinine clearance is below 30 milliliters/minute [31]. Because worsening kidney function is associated with a major risk of bleeding [32], frequent monitoring is required in older individuals taking VKA. The choice of VKA by prescribers in severe heart failure or heart rhythm disorders could be due to the fact that DOAC are contraindicated in valvular AF. The choice of VKA could also be associated with patient frailty [33]. The low rate of kidney excretion of apixaban (27%) compared to rivaroxaban (33%) and dabigatran (80%) [30] could explain the fact that apixaban was the most commonly prescribed DOAC in our study, followed by rivaroxaban and dabigatran. This was previously found by Manckoundia et al. [19]. In parallel, the liver is responsible for the production of most factors involved in coagulation [34]. Thus, in active chronic liver diseases or cirrhosis, DOAC and VKA should be used with caution. Apixaban and rivaroxaban are mainly cleared through the liver (75% and 65%, respectively), while only 20% of dabigatran is eliminated by the liver (80% by the kidney) [35]. In addition, plasma-binding protein reaches 95% for rivaroxaban, 85% for apixaban, and is lower for dabigatran (35%). This may result in higher levels of free fraction of the medication when synthesis of albumin in the liver is reduced. In addition, apixaban and rivaroxaban are metabolized by cytochrome P450 enzymes, whose activity is diminished in liver disease [35]. Some observational studies have shown that DOAC are more effective and safer than traditional anticoagulants [36,37]. The European Medicines Agency recommend using the Child–Pugh score to guide the prescription of DOAC [38]. This guideline does not recommend DOAC use in patients with Child–Pugh grade C liver disease or with any coagulopathy-associated liver disease. For patients taking VKA, the management of drug dosage is challenging considering that warfarin, which has plasma protein binding nearing 99%, is eliminated by the liver through cytochrome P450 and the international normalized ratio (INR) increases in cirrhosis. The INR is not well defined in this population, resulting in a higher risk of thrombotic events when the dosage is suboptimal and an increased bleeding risk when it is supratherapeutic [39]. In our study, the patients with liver disorder were not sufficiently represented, with only 12 in total: 7 in the DOAC group and 5 in the VKA group.

Heart failure and AF are frequently associated and, when both entities coexist, it results in a high risk of thromboembolism. In AF, DOAC are associated with the reduction of thromboembolic events. However, it seems that individuals with heart disease treated with DOAC, especially in cases of intracardiac thrombosis, have a higher risk of ischemic stroke than those treated with warfarin, explaining the rate in our study [40].

The combination of Alzheimer’s disease and DOAC could be due to the conclusion made in some studies that DOAC could protect against the progression of dementia by targeting thrombin, which is an early pathological hallmark of Alzheimer’s disease [41].

Other factors or diseases associated with frailty, such as polypathology, which is not included in the RCD; disabling stroke; ischemic peripheral or coronary artery diseases; and diabetes [42] were not prevalent in either of the groups. This is surprising, especially because frail individuals have a higher risk of falling [43] and because their comorbidities contribute to a higher risk of drug-related adverse events. Kim et al. compared the outcomes of DOAC vs. VKA by frailty levels and found that apixaban was associated with lower rates of adverse events across all frailty levels [44].

Compared to the 2017 study, there were significantly more individuals in the VKA group with VTE than in the DOAC group, despite DOAC being safer and more convenient [45]. One explanation for this could be the complexity of prescribing DOAC, requiring the prescriber to have expertise in VTE. This result was also highlighted in 2017 in a Danish nationwide cohort study [46], in which Sindet-Pedersen et al. found that the prescription of DOAC was increasing, but individuals were more likely to be treated with VKA when they had one of the following: previous VTE, chronic kidney disease, liver disease, cancer, or thrombophilia.

In our study, AF was an independent factor associated with the prescription of DOAC, as confirmed in the multivariate analysis, meaning that the current guidelines [20] are effective and respected. According to these guidelines, DOAC are the preferred antithrombotic therapy in older individuals [18,20,47]. It is already known that DOAC are associated with a significant reduction in the risk of stroke, intracranial hemorrhage, and mortality [18]. They are associated with an increased risk of gastrointestinal bleeding but have an equal risk of major bleeding compared to warfarin, leading to a favorable risk–benefit profile [48,49]. Since our first study in 2017, other studies have confirmed the safety and effectiveness of DOAC vs. VKA in older patients with AF [50].

Regarding medications, most cardiovascular drugs, such as beta-blockers, calcium-channel blockers, angiotensin-converting enzyme inhibitors, thiazide diuretics, spironolactone, and nitrate derivatives, were prescribed at similar rates in the two groups. The two drugs that were confirmed in the multivariate analysis as being associated with VKA rather than DOAC prescription were furosemide and fibrates. PPI and antirheumatic treatments were surprisingly the determining factors for the prescription of DOAC. The use of PPI could be linked with the fear of the risk of a gastrointestinal bleed [51]. The association of antirheumatics could be due to the fact that some antirheumatic agents, including non-steroidal drugs, increase the risk of bleeding when associated with warfarin [52]. In our study, there was no significant difference between the two groups in terms of mortality rate: 8.6% of individuals were deceased in the DOAC group and 9.41% in the VKA group.

While this study has the advantage of analyzing real-life data from the MSAB and comparing the habits of prescribers in France concerning oral anticoagulation four years after the initial study was performed, one of the limitations still lies in its retrospective design and the short timeframe analyzed (i.e., one month). Still, about 4275 individuals aged 80 years and older were included. Another limitation is related to the extrapolation of anticoagulant indications from declared RCD because we did not have access to complete medical records. Indeed, anticoagulant indications which are not RCD or which are not declared as RCD are, therefore, not known. Furthermore, we did not have access to the results of the patients’ laboratory tests, which would have allowed us to estimate renal and liver functions. Additionally, while AF is always declared as an RCD, certain VTE events might not have been recorded if they were considered non-serious by prescribers. The lack of reporting for VTE events could explain the low rate (3.92%) of VTE in the whole sample. Again, because we did not have access to complete medical records, we were unable to identify the precise reasons for maintaining VKA, which is contrary to the current guidelines in the absence of contraindications.

Our results are based on regional data and cannot be generalized to all populations. However, the database used for this study is nonetheless very valuable as it can be used to generate pertinent real-life studies and it makes it possible to conduct a comparison study several years after the initial study has been conducted.

## 5. Conclusions

In this sample composed of individuals aged 80 years and older, DOAC were more frequently prescribed than VKA. Although the mean age and mean number of drugs per prescription were similar in the two groups, individuals who received VKA had more severe comorbidities (RCD), especially severe chronic nephropathy and severe heart failure or heart rhythm disorder. By comparing this study to the one carried out by our team in 2017, we are able to see how prescribers’ practices have shifted in accordance with the current guidelines. With aging, kidney function worsens, increasing the bleeding risk. There is a need to promote studies assessing the benefit–risk profile of DOAC vs. VKA in patients with severe kidney failure so that new data can be used to adapt the use of certain drugs.

## Figures and Tables

**Table 1:** Comparison of age, sex, existence of one or more registered chronic diseases (RCD), duration of oral anticoagulant prescription, medical specialty of the prescriber, frequencies of atrial fibrillation (AF) and venous thromboembolism (VTE), and mean number of medications per prescription in the subjects prescribed direct oral anticoagulants (DOAC) or vitamin K antagonists (VKA), using bivariate logistic regression.

Variables		DOAC Group (N = 2883)	VKA Group (N = 1392)	OR [95% CI]	*p*
		Mean ± SD or % (N)	Mean ± SD or % (N)		
Mean age (years)		87.58 ± 4.52	87.85 ± 4.64	0.98 [0.97–1.00]	0.075
Age range (years)	80–84	28.27 (815)	27.37 (381)	Reference	
	85–89	38.64 (1114)	35.99 (501)	1.04 [0.88–1.22]	0.186
	90–94	25.42 (733)	27.8 (387)	0.89 [0.75–1.05]	0.105
	95–99	7.18 (207)	8.12 (113)	0.86 [0.66–1.11]	0.303
	>100	0.49 (14)	0.72 (10)	0.65 [0.29–1.49]	0.462
Sex	Women	54.98 (1585)	46.98 (654)	Reference	
	Men	45.02 (1298)	53.02 (738)	0.73 [0.64–0.83]	<0.001
RCD	No RCD	8.53 (246)	6.32 (88)	Reference	
	≥1 RCD	91.47 (2637)	93.68 (1304)	0.72 [0.56–0.93]	0.014
Mean number of RCD		1.78 ± 1.12	2.05 ± 1.21	0.82 [0.78–0.86]	<0.001
Mean number of drugs/prescriptions		7.40 ± 2.804	7.69 ± 2.98	0.97 [0.94–0.99]	0.002
Anticoagulant duration	Initiation	66.11 (1906)	19.61 (273)	Reference	
	Refill	33.89 (977)	80.39 (1119)	0.12 [0.11–0.15]	<0.001
Prescriber specialty	General Practitioner	86.78 (2502)	92.03 (1281)	0.57 [0.46–0.71]	<0.001
	Other specialties	13.22 (381)	7.97 (111)	Reference	
Anticoagulant indication	AF	41.55 (1198)	47.20 (657)	0.80 [0.70–0.90]	0.001
	VTE	2.53 (73)	6.75 (94)	0.36 [0.26–0.49]	<0.001
Deceased		8.60 (248)	9.41 (131)	0.91 [0.73–1.13]	0.389
Anticoagulation switch		8.68 (246)	1.14 (16)	8.02 [4.83–13.36]	<0.001

DOAC: direct oral anticoagulant, VKA: vitamin K antagonist, N: number, OR: odds ratios, CI: confidence intervals, SD: standard deviation, RCD: registered chronic diseases, AF: atrial fibrillation, VTE: venous thromboembolism.

**Table 2:** Comparison of registered chronic diseases and drugs in the individuals of both groups using bivariate analysis. We report only significant differences.

Variables		DOAC Group (N = 2883)	VKA Group (N = 1392)	OR [95% CI]	*p*
		% (N)	% (N)		
RCD	Alzheimer’s disease	7.49 (216)	4.31 (60)	0.56 [0.41–0.75]	<0.001
	Severe heart failure or heart rhythm disorders	56.50 (1629)	68.03 (947)	1.64 [1.43–1.87]	<0.001
	Severe hypertension	17.31 (499)	21.62 (301)	1.32 [1.12–1.55]	0.001
	Severe chronic nephropathy and/or PNS	1.38 (40)	3.59 (50)	2.65 [1.74–4.03]	<0.001
	Severe chronic respiratory failure	2.77 (80)	4.17 (58)	1.52 [1.08–2.15]	0.021
	Illnesses not on the list	8.91 (257)	14.08 (196)	1.67 [1.37–2.04]	<0.001
Drug	Furosemide	48.53 (1399)	55.75 (776)	1.34 [1.18–1.52]	<0.001
	Digoxin	9.23 (266)	12.07 (168)	1.35 [1.10–1.66]	0.005
	Other antiarrhythmic drugs	14.08 (406)	11.71 (163)	0.81 [0.67–0.98]	0.035
	Proton pump inhibitors	43.95 (1267)	39.44 (549)	0.83 [0.73–0.95]	0.006
	Fibrates	2.32 (67)	3.88 (54)	1.70 [1.18–2.44]	0.006
	Antirheumatics	1.60 (46)	0.65 (9)	0.40 [0.20–0.82]	0.009
	Statins	26.99 (778)	30.60 (426)	1.19 [1.04–1.37]	0.015
	Nitrate derivatives	4.65 (134)	6.39 (89)	1.40 [1.06–1.85]	0.019
	Calcium channel blockers	22.06 (636)	24.86 (346)	1.17 [1.01–1.36]	0.044

RCD: registered chronic diseases, DOAC: direct oral anticoagulant, VKA: vitamin K antagonist, N: number, OR: odds ratio, CI: confidence interval, PNS: primitive nephrotic syndrome.

**Table 3:** Comparison of selected variables in the individuals prescribed direct oral anticoagulants (DOAC) or vitamin K antagonists, using multivariate logistic regression. The results are interpreted with reference to the DOAC group.

Variables	OR [95% CI]	*p*
Male sex	0.80 [0.69–0.93]	0.003
Refill prescriptions	0.12 [0.15–0.11]	<0.001
General practitioner as prescriber	0.72 [0.56–0.94]	0.014
Atrial fibrillation	1.30 [1.06–1.60]	0.011
VTE	0.34 [0.23–0.50]	<0.001
Severe heart failure or heart rhythm disorders	0.68 [0.55–0.86]	0.001
Alzheimer’s disease	1.83 [1.30–2.57]	0.001
RCD	0.96 [0.86–0.99]	0.036
Severe chronic nephropathy and/or PNS	0.31 [0.19–0.50]	<0.001
Anticoagulation switching	2.36 [1.34–4.16]	0.003
Beta-blockers	1.12 [0.96–1.30]	0.149
Other antiarrhythmic drugs	1.20 [0.96–1.49]	0.112
Furosemide	0.76 [0.66–0.89]	0.001
Nitrate derivatives	0.78 [0.57–1.08]	0.135
Fibrates	0.54 [0.36–0.83]	0.004
Other cholesterol and triglyceride regulator	0.58 [0.31–1.09]	0.089
Calcium channel blockers	0.85 [0.71–1.01]	0.065
PPI	1.21 [1.04–1.40]	0.015
Heparins	0.51 [0.30–0.88]	0.012
Antirheumatics	2.59 [1.19–5.61]	0.016

OR: odds ratio, CI: confidence interval, VTE: venous thromboembolism, RCD: registered chronic diseases, PNS: primary nephrotic syndrome, PPI: proton pump inhibitors.

## Data Availability

The data presented in this study are available from the corresponding author upon request. The data are not publicly available.
